# Global health research collaborations: lessons from the Fogarty International Center

**Published:** 2011-01-10

**Authors:** Roger I Glass

**Affiliations:** 1 Fogarty International Center, National Institutes of Health, Bethesda, Maryland 20892-2220, USA

## 

The Fogarty International Center at NIH has the mission of supporting training and research in global health with a focus on problems in low and middle income countries. Our programs provide research opportunities in both infectious and chronic diseases that link investigators and institutions in the United States with groups in the developing world. The value of these long term investments is evident by their ability to support cutting edge research while training local investigators to develop independent streams of funding and support. The presentation will provide examples of our activities that might have application within the network of the Institut Pasteur.

